# Utilization of Crumb Rubber and High-Volume Fly Ash in Concrete for Environmental Sustainability: RSM-Based Modeling and Optimization

**DOI:** 10.3390/ma14123322

**Published:** 2021-06-16

**Authors:** Mugineysh Murali, Bashar S. Mohammed, Isyaka Abdulkadir, M. S. Liew, Wesam Salah Alaloul

**Affiliations:** 1 Civil and Environmental Engineering Department, Universiti Teknologi PETRONAS, Bandar Seri Iskandar 32610, Malaysia; mugineysh_24401@utp.edu.my (M.M.); isyaka_18000638@utp.edu.my (I.A.); shahir_liew@utp.edu.my (M.S.L.); wesam.alaloul@utp.edu.my (W.S.A.); 2 Civil Engineering Department, Bayero University, Kano 700241, Nigeria

**Keywords:** crumb rubber (CR), high-volume fly ash (HVFA), response surface methodology (RSM), optimization

## Abstract

Waste tire and fly ash (FA) are two waste materials whose disposal and rapid rate of accumulation are among the pressing sources of concern and threat to the environment. Although much research exists on the use of these materials in cementitious composites, very little literature is available on the effectiveness of combining them in high volumes for concrete production. This work aimed to utilize crumb rubber (CR) from waste tires as a partial replacement of fine aggregate at 15%, 22.25%, and 30% by volume, and high-volume fly ash (HVFA) replacement of cement at 50%, 60%, and 70% (by weight of cementitious materials) to produce high-volume fly ash–crumb rubber concrete (HVFA–CRC). Using the central composite design (CCD) option of the response surface methodology (RSM), 13 mixes were produced with different combinations and levels of the CR and FA (the input factors) on which the responses of interest (compressive, flexural, and tensile strengths) were experimentally investigated. Furthermore, the composite influence of CR and HVFA on the workability of the concrete was assessed using the slump test. The results showed a decline in the mechanical properties with increasing replacement levels of the CR and HVFA. However, up to 22.25% and 60% of CR and HVFA replacements, respectively, produced a structural HVFA–CRC with a compressive strength of more than 20 MPa at 28 days. Response predictive models were developed and validated using ANOVA at a 95% confidence level. The models had high R^2^ values ranging from 95.26 to 97.74%. Multi-objective optimization was performed and validated with less than 5% error between the predicted and experimental responses.

## 1. Introduction

To achieve the noble aim of environmental sustainability, governments and other relevant organizations are increasingly focused on finding more efficient ways of curtailing the depletion and degradation of natural resources. One such measure is controlling the amount of waste generation and disposal. Waste tire generation and disposal are among the most pressing environmental challenges that need to be addressed. The world is experiencing a rapid increase in automobile production to cope with the rising population and transportation needs [1]. This leads to the rise in waste tire generation at a rate that far exceeds its recycling and reuse. More than 1 billion tires are produced globally every year [2]. It is projected that the annual waste tire generation could reach 5 billion by 2030 [3]. The United States of America leads as the country with the highest annual waste tire generation with 270 million tires, followed by Japan with 110 million tires [2]. The excess waste tire ends up being disposed of at landfills and often indiscriminately in waterways and other inappropriate sites. Many countries have banned the disposal of waste tires in landfills because of their rapid rate of accumulation, risk of uncontrollable fire outbreak, toxic smoke emission, etc. [3]. It is against this backdrop that researchers investigate CR as a replacement for fine aggregate in concrete. This practice has a double advantage for the environment due to solving the waste disposal challenge and natural aggregate depletion [4]. The use of CR in concrete enhances the deformation capacity, impact resistance, energy absorption, resistance to cyclic freezing and thawing, decrease in water absorption, and chloride permeability [2,3,4,5,6].

Another threat to the environment is fly ash (FA) generation. FA is a byproduct of coal combustion from power plants [7], which is used as a supplementary cementitious material in cementitious composites, geopolymer concrete, filling material in rubber and plastic, etc. However, the rate of its generation is far beyond its current use. It is considered a waste material, with 600 million tons generated annually, 80% of which is dumped in ash dams and landfills [8]. This poses a threat to the environment. ASTM C168 classified two types of FA for use in Portland cement concrete based on their chemical composition. Low-calcium FA (class F) and high-calcium FA (class C). Class F FA is the most widely used type of FA in concrete and geopolymer mortars due to its high reactivity and performance as a pozzolanic material. However, class C FA is associated with high CaO content, which causes a lot of cement instability and hence hinders its utilization [9]. However, due to the recent rise in the generation of class C FA as a result of the combustion of sub-bituminous coal in power plants, research on its utilization has been intensified [9]. The use of FA in concrete was limited to 20–25% by weight of cementitious materials [10]. Hence, finding ways of utilizing more of the FA in alkali-activated aluminosilicate materials is needed in order to drastically reduce the dumping of this waste and its related consequences [11,12]. For this reason, the first use of HVFA in the production of structural concrete was proposed in the early 1980s by the Canadian Centre for Mineral and Energy Technology (CANMET) [10]. HVFA concrete contains a minimum FA of 50% by weight of cementitious materials [13]. Recently, researchers utilized up to 70% HVFA in cementitious composites, as reported in the following literature: Sun et al. [14] investigated the effect of using 40–70% HVFA on the compressive strength and hydration behavior of concrete. Similarly, Rashad [15] investigated the high-temperature behavior of HVFA paste containing 70% FA cement replacement and micro-sized metakaolin subjected to high temperatures. HVFA jointing mortar containing 0–70% FA replacement of cement was investigated by Posi et al. [16]. From previous research findings, the use of HVFA in concrete enhances its workability, reduces drying shrinkage, lowers the heat of hydration, and enhances elevated temperature performance. Furthermore, it cuts down on the CO_2_ emissions and cost of concrete production due to excessive cement use [12,17].

Although the use of CR and FA in concrete has evolved for more than three decades, combining these two green materials in concrete has not been thoroughly investigated. This is evident from the scanty literature available on the topic. The earliest literature available on combined FA and CR in concrete was by Hilal [18]. He investigated the effect of different CR sizes and contents on the hardened properties of self-compacting concrete when utilizing 30% FA as a replacement for cement. CR replacement levels of 5 to 25% of fine aggregate were considered in the research. The most recent work utilizing FA and CR in concrete is by Fauzan [19]. The work compared the effect of CR on the mechanical properties of normal and FA concrete. Levels of 5, 10, 15, and 20% CR replacement of fine aggregate and 15% FA replacement of cement were considered for that study. These two cited works used less than 50% FA (30 and 15%, respectively), and the investigated concrete was therefore not considered to be HVFA concrete.

Al-Fakih et al. [20] developed relationships for the compressive strength of a concrete masonry wall made with 10% CR replacement of fine aggregate and 56% FA replacement of cement. Furthermore, the dual effect of nano-silica and CR on HVFA roller-compacted concrete was investigated by Adamu et al. [21] and Ameli et al. [22]. Similarly, Bisht and Ramana [23] evaluated the mechanical and durability properties of Portland pozzolana cement (PPC) concrete containing 4–5.5% crumb rubber replacement of fine aggregate. The PPC cement used was an industrially produced blend of FA and Portland cement. Although these studies considered HVFA and CR in one way or the other, they fell short in addressing the issue that this research wished to address: the use of HVFA and CR in structural concrete.

It is obvious from the above that not much work has been done on the use of HVFA together with CR in concrete. More work in the area of engineered cementitious composite (ECC) and geopolymer concrete utilizing HVFA and CR are available than in structural concrete. This research aimed to assess the properties of a green HVFA–CRC that was produced using a high amount of waste materials (CR and FA) for environmental sustainability and cost reduction. The experiment involved the RSM tool to model and optimize the input factors (CR and FA) to yield a concrete of desirable quality in fresh and hardened states. This research will be the first to utilize HVFA and CR as replacements of cement and fine aggregate, respectively, and develop response predictive models of the mechanical properties of the composite using the RSM tool. The significance of this research lies in the potential solution to the environmental degradation caused by waste tires and FA generation and disposal, as well as the depletion of the natural raw materials.

## 2. Materials and Methods

### 2.1. Materials

**Figure 1:**
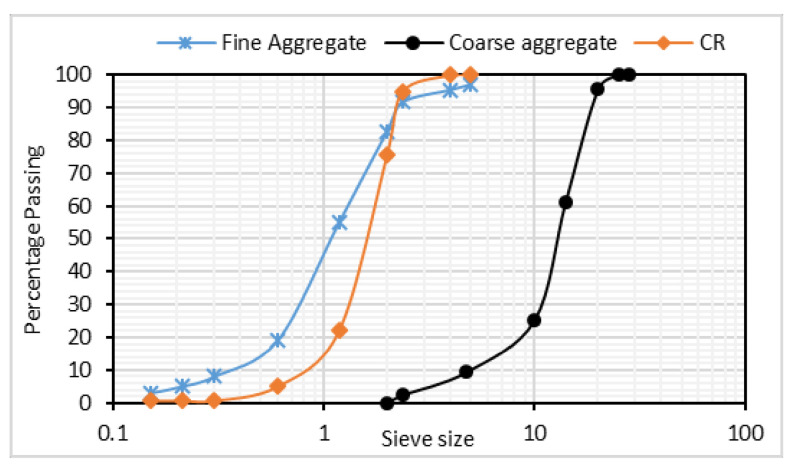
Particle Size distribution curves for Aggregates and CR used.

**Figure 2:**
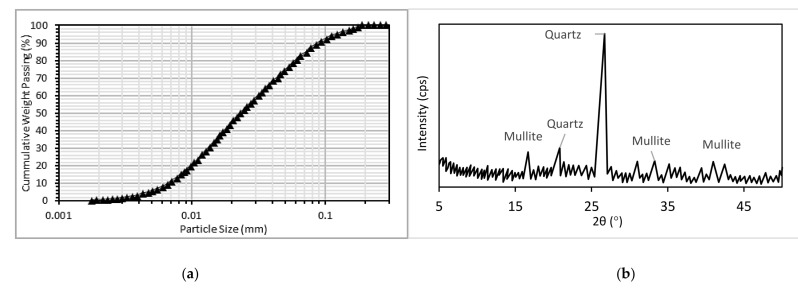
(**a**) Particle size distribution and (**b**) XRD pattern of the FA.

Type I ordinary Portland cement (OPC) satisfying the specifications of ASTM C150 and with a specific gravity of 3.16 was used. Low calcium FA having a specific gravity of 2.38, a specific surface area of 380 m^2^/kg, a loss on ignition (LOI) of 1.85, and satisfying the specifications of ASTM C618 was used for cement replacement at 50, 60, and 70% by weight of total binder content. Table 1 presents the chemical composition of the cement and fly ash used. The chemical composition was determined using X-ray fluorescence (XRF) analysis. The fine aggregate used was river sand with a specific gravity of 2.65 and a crushed coarse aggregate with 20 mm nominal size and specific gravity of 2.60 was used. A CR having a specific gravity of 1.10 and particle size as shown in Figure 1 for the grading curves of fine aggregate, coarse aggregate, and CR was used for the fine aggregate replacement at 15, 22.5, and 30% by volume. The grading curves for the aggregates and CR are presented in Figure 1. The grading curve was plotted using the data from the sieve analysis performed on the materials based on the provisions of ASTM C136/C136M. The particle size distribution of the FA was determined using a Horiba (LA960) particle size analyzer (Horiba, Kisshoin, Minami-ku Kyoto, Japan) and the curve is presented in Figure 2a. The XRD of the FA was performed using a Bruker X-ray diffractometer (Bruker, Billerica, MA, USA) and the result showing the amorphous content is presented in Figure 2b. A constant water–binder ratio (W/B) of 0.40 was used for all mixes.

### 2.2. Response Surface Methodology (RSM) and Mix Proportioning

Using the RSM to achieve the aim of this research, the two independent variables (input factors) that were considered were the CR and FA at three replacement levels of 15, 22.25, and 30% of fine aggregate and 50, 60, and 70% of cement, respectively. Thirteen experimental runs were generated using the rotatable central composite design (CCD) option of RSM. As shown in Table 2, the mixes had varying combinations and levels of the input factors and five randomized duplications for each variable. The duplicate mixes were to ensure the effectiveness of the experiment and guard against possible deviations [24]. The RSM measures the influence of the interaction between the input factors on the responses. The responses that were considered were the compressive strength, flexural strength (modulus of rupture), and splitting tensile strength.

In order to produce concrete with significant mechanical strength, the quantities of materials required for M50 concrete were adopted from Soutsos et al. [25]. Hence, a water–binder ratio (W/B) of 0.40 was used. The quantities of materials required to produce the test samples are shown in Table 2.

### 2.3. Sample Preparation and Testing

#### 2.3.1. Mixing and Casting

The samples were made from well-mixed HVFA–CRC that was prepared following the specifications of BS 1881: Part 125:1986. The fine aggregate, coarse aggregate, and CR were dry-mixed for 25 s in a concrete mixer. Half of the mixing water was then added and mixed for 1 min. This was followed by adding the cement and FA and mixing for 1 min. The remaining water was added and mixed until the fresh HVFA–CRC looked homogenous and consistent. To ensure the uniformity of the mix, further mixing was done manually using a hand trowel.

The fresh concrete was cast into molds for the relevant test samples. Lightly oiled steel molds were used for casting the samples to allow for easy demolding. For the compressive strength test, 100 mm cube samples were cast. Beam prisms with dimensions 500 mm × 100 mm × 100 mm were cast for the flexural test and 300 mm height by 150 mm ø cylinder samples were made for the splitting tensile strength test. The samples were left for 24 h before demolding, labeled, and cured in water at 20 °C and 95% relative humidity for the required number of days.

#### 2.3.2. Slump Test

**Figure 3:**
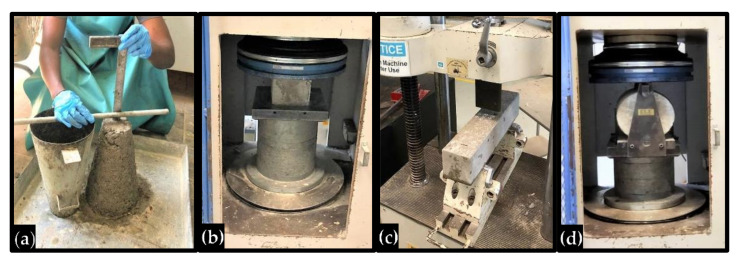
Tests on the HVFA–CRC: (**a**) slump test, (**b**) compressive strength test, (**c**) flexural test, and (**d**) splitting tensile test

The test was performed following the requirements of BS EN 12350-2: 2009 using a slump cone with upper and lower opening diameters of 100 mm and 200 mm, respectively. The cone was filled with the concrete in three layers and compacted using a 16 mm diameter, 600 mm long tamping rod by tamping the concrete 25 times. The mold was removed and the height difference between the cone and the slumped concrete was recorded as the slump of the concrete, as shown in Figure 3a.

#### 2.3.3. Hardened Properties Tests

The compressive strength (CS) test was performed based on the specifications of BS EN 12390-3:2019 at 7, 14, and 28 days of curing. The samples were tested by subjecting them to a uniaxial compressive load by means of a 3000 kN universal testing machine (UTM), as shown in Figure 3b. The average of three results is reported as the compressive strength of the mix for that particular curing duration.

A three-point flexural test was conducted following the specifications of BS EN 12390-5:2019 using a 200 kN UTM, as shown in Figure 3c. Three samples were tested for each mix at 28 days of curing. The test data was obtained through a computer data acquisition system attached to the UTM. The flexural strength (FS) and mid-span deflection of the samples were determined from the test data.

The splitting tensile test was performed using a 3000 KN UTM in accordance with the provisions of BS EN 12390-6:2019, as shown in Figure 3d. Using the 300 mm by 150 mm ø cylinders, the splitting tensile strength (STS) of the mixes was determined at 28 days of curing.

## 3. Results and Discussions

Table 3 presents the results from the tests done on the HVFA-CRC.

### 3.1. Slump of the HVFA–CRC

**Figure 4:**
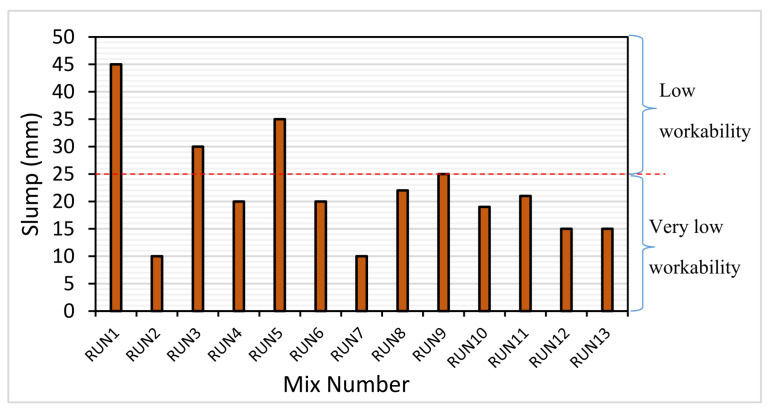
Slump of the HVFA–CRC.

The slump test result is shown in Figure 4. It can be seen from the graph that a higher FA replacement led to higher workability. This was seen from mixes with 70% FA having a higher slump than the others with the same CR content but a lower FA content. For example, RUN1 (15% CR, 70% FA) had 200% and 80% higher slump than RUN13 (15% CR, 50% FA) and RUN 9 (15% CR, 60% FA), respectively. In the same vein, the mixes having the lowest FA content (RUN 2 and RUN7) had the lowest workability.

**Figure 5:**
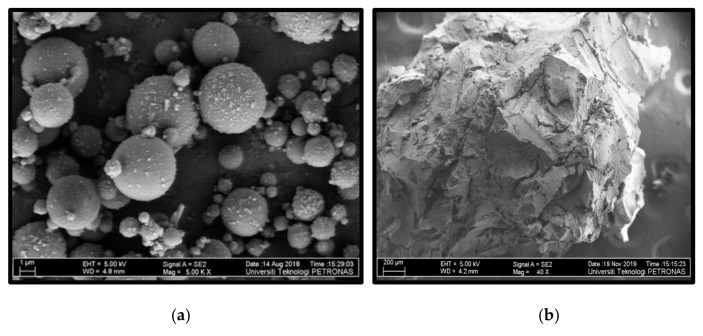
FESEM images showing the (**a**) spherical shape of the FA particles [29] and (**b**) the rough surface texture of the CR.

The enhanced workability with increasing FA replacement was due to the spherical morphology of the FA particles. The nature of the FA particles is shown in the FESEM image in Figure 5a. The FA particles behaved like small ball bearings in the mix, which Khed et al. [26] called “the ball bearing effect.” This effect reduces the viscosity and yield stress of the mix, leading to higher workability. Furthermore, the reduction in the higher density cement particles by the FA led to a decrease in the yield stress, allowing for a greater fluidity of the mix. Moreover, the tendency of cement particles to trap water through flocculation is reduced with the FA replacement, as explained by Abdulkadir et al. [27].

On the other hand, the workability of the HVFA–CRC mixes reduced with increasing CR replacement. This was observed from the slump for mixes with the same FA content but different CR content. RUN3 (30% CR, 70% FA) had a 16.6% and 50% lower slump compared with RUN5 (22.5% CR, 70% FA) and RUN1 (15% CR, 70% FA), respectively. This trend was observed across all mixes with the same FA content but different CR content. The negative influence of the CR on the workability of cement composites has been reported by previous researchers, such as Assaggaf et al. [4] and Siddika et al. [28]. This behavior was due to the rough texture of the CR particles, as presented in Figure 5b, which led to higher internal friction that required more energy for the fresh concrete to flow.

The slump for all the 13 mixes ranged from 20–45 mm, which were classified under the “very low” to “low” workability categories. The three mixes (RUN1, RUN3, and RUN6) in the “low workability” class had 70% FA, while the rest of the mixes fell into the “very low workability” class. The low slump was due to the low W/B (0.40) and the absence of a superplasticizer in the mix. As stated earlier, the low W/B was used to achieve a high concrete strength. The workability of the mixes can be greatly enhanced if a superplasticizer is used, as reported from previous research on CRC [4].

### 3.2. HVFA–CRC Compressive Strength

**Figure 6:**
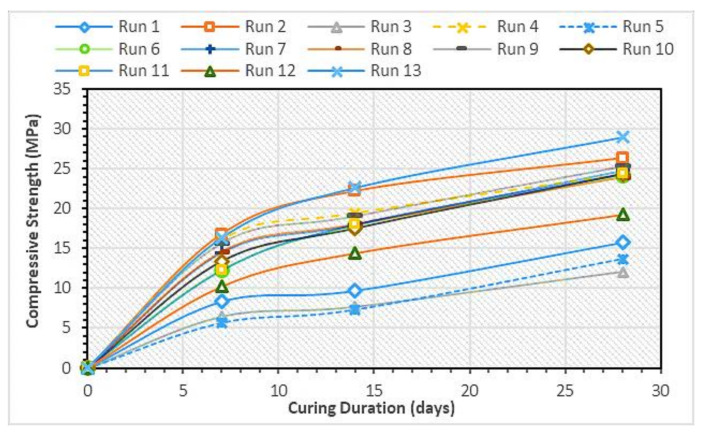
Rate of compressive strength development for the HVFA–CRC.

The rate of development for the compressive strength of the HVFA–CRC is shown in Figure 6. Generally, the strength increased with increased curing time. For all mixes, the rate of strength gain was higher in the first two weeks than in the latter part of the curing duration. This was due to the nature of the hydration reaction, which proceeds faster in the early stages because of the presence of the higher water availability for the reaction than in the later stages. One thing to note, however, is that the rate of strength gain was lower with the higher FA content. In other words, mixes having a lower FA content gained strength faster. This is ascribed to the reduction in the cement due to the FA replacement, which led to a lower amount of cement hydration products, such as the calcium silicate hydrate (C-S-H) gel responsible for the strength. This led to a lower rate of strength gain for mixes having a higher FA content. From the graph, the trends show that the strength development was likely to continue beyond the 28 days. This is credited to the pozzolanic nature of the class F FA used. In the pozzolanic reaction, the FA reacts with the Ca(OH)_2_ produced from the cement hydration to form secondary C-S-H gel in addition to the one generated during the primary cement hydration. The reaction, although slow at the beginning, goes on for a long time, leading to increased strength at later stages of the composite. This behavior is consistent with the findings of previous research on FA replacement of cement in composites [9,11,30,31].

**Figure 7:**
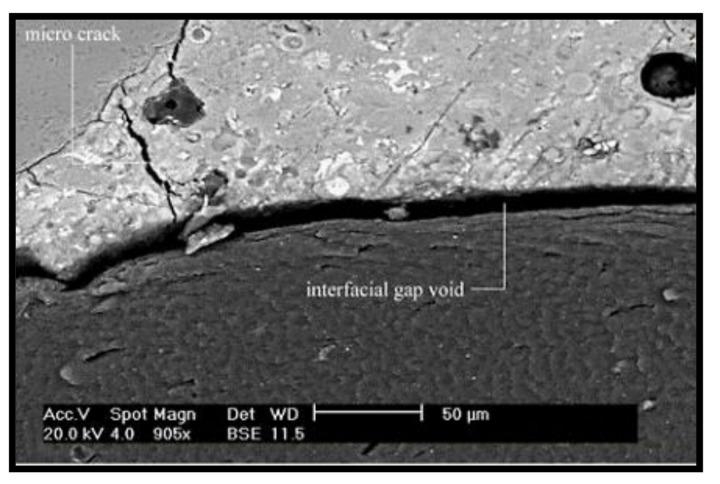
SEM image showing the lack of proper bonding between the CR and hardened cement matrix at the interface [32].

On the other hand, because strength development is a chemical process, CR does not have any influence, as it participates at a physical level. It was observed that the compressive strength of the concrete decreased with an increase in the CR replacement. The main reason behind the decrease in compressive strength with increasing CR content is due to the lack of proper bonding between the CR and the hardened cement matrix at the interface, as reported by Najim and Hall [32] and as depicted in Figure 7. This poor bonding was because of the hydrophobic nature of CR particles, which repel water during mixing. Moreover, the CR led to increased porosity of the composite, thereby negatively affecting the strength. Furthermore, the soft nature and lower elastic modulus of the CR compared with the fine aggregate particles contributed to the lower strength of the concrete, serving as weak points within the composite, as reported in previous works [2,4,19,27].

**Figure 8:**
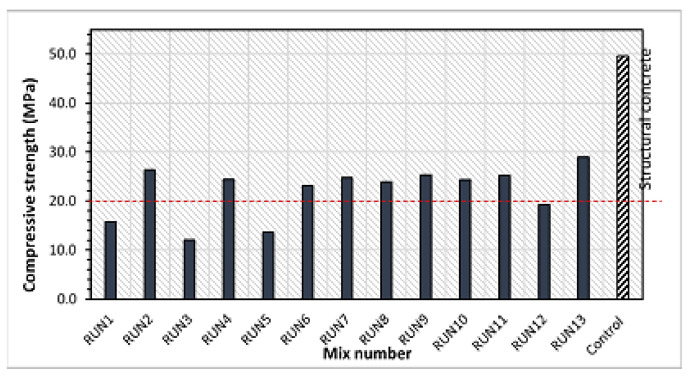
Compressive strength of the HVFA–CRC mixes at 28 days.

Figure 8 displays the 28-day compressive strength of the HVFA–CRC mixes. It can be observed that nine out of the thirteen HVFA–CRC mixes had a strength of more than 20 MPa at 28 days, as indicated by the red line. The mixes with compressive strength below the required minimum strength for structural concrete (20 MPa) were RUN1, RUN3, and RUN5 (all having 70% FA replacement) and RUN12 (CR: 30%, FA: 60%). The lower W/B of 0.40 used contributed to attaining relatively high compressive strength at high volumes of these two waste materials (CR and FA). As reported by Gang et al. [9], high-volume fly ash increases the effective W/B ratio, thereby enhancing the degree of hydration of the composite. Hence, the use of 0–22.25% CR, together with HVFA of up to 60%, can yield a concrete of appreciable strength that can be used for structural applications.

**Figure 9:**
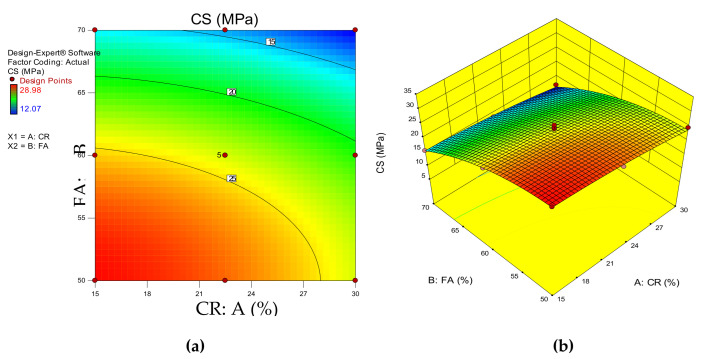
HVFA–CRC 2D and 3D response surface graphs for CS.

Figure 9a,b depict the 2D and 3D response surface diagrams of the HVFA–CRC. These plots depict the influence of the interaction between the input variables on the response (compressive strength). The red regions show the areas of high compressive strength intensity. Meanwhile, the green and blue regions indicate areas of medium and low compressive strength values, respectively. As can be observed, the area bounded by the 25 MPa contour line and the graph axes (at 61% FA and 28% CR) had the highest response. Any combination of the variables below these two boundary values (61% FA and 28% CR) will yield an HVFA–CRC with more than 25 MPa. The response was lower for any combination of the variables above the stated boundary.

### 3.3. Flexural Performance of the HVFA–CRC

**Figure 10:**
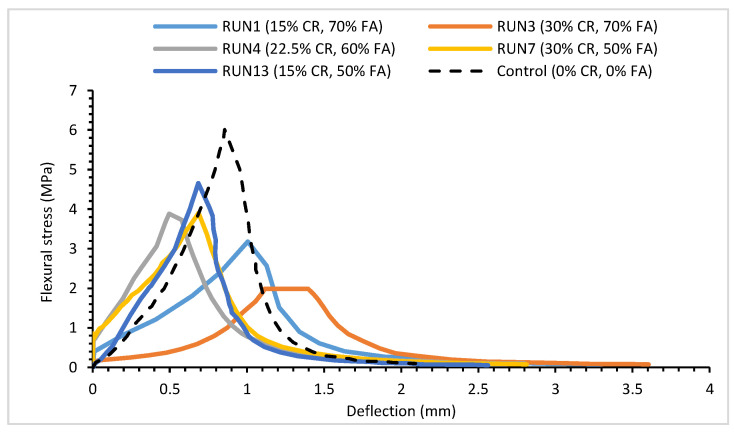
Flexural stress–strain graph for certain HVFA–CRC mixes.

**Figure 11:**
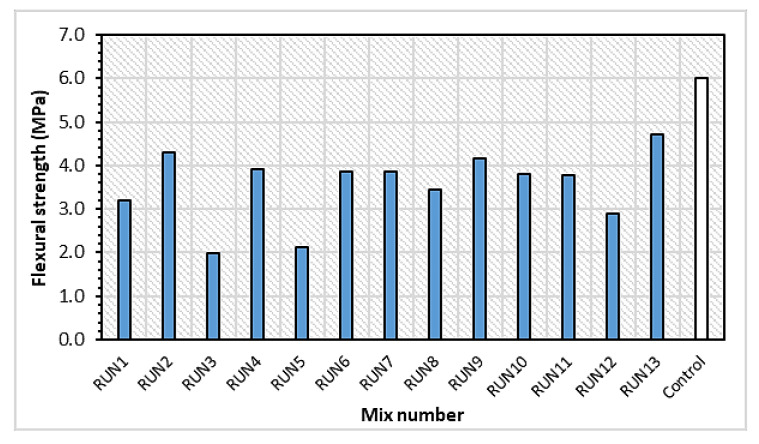
Flexural strength of the HVFA–CRC mixes at 28 days.

Figure 10 and Figure 11 show the flexural stress–strain curves for some selected mixes and the flexural strength for all the mixes at 28 days, respectively. A control mix having 0% CR and 0% FA replacements was produced for the purpose of comparison. As expected, the combined effect of CR and HVFA led to a lower flexural strength of the composite but positively enhanced its ductility. As depicted in Figure 11, RUN13 with the lowest CR and FA content of all the mixes had a 21.8% lower flexural strength compared with the control. However, it had about a 22% higher deflection compared with the control. In the same vein, the mix having the highest CR and FA contents of 30% and 70%, respectively (RUN3) had the lowest flexural strength of all the 13 mixes. However, it exhibited the highest deflection capacity of 3.6 mm, which was 71.4% higher than the control mix.

The lower flexural strength of the concrete with increasing CR and HVFA was attributed to the similar reason stated for compressive strength. However, the enhanced ductility was attributed to (1) the flexible nature and low elastic modulus of the CR particles, which can easily bend under load, and (2) the toughness reduction effect of the FA. At 28 days, most of the HVFA remained unreacted within the composite serving as a filler, thereby refining the pore structure of the composite and enhancing the density and increasing the deformation capacity. Similar findings regarding the effect of CR in concrete have been reported in previous research [2,33]. Similarly, higher ductility is associated with a higher energy absorption capacity. Hence, the use of these two waste materials in a high volume can be beneficial in structures where fatigue failure is common and energy absorption is required [4].

**Figure 12:**
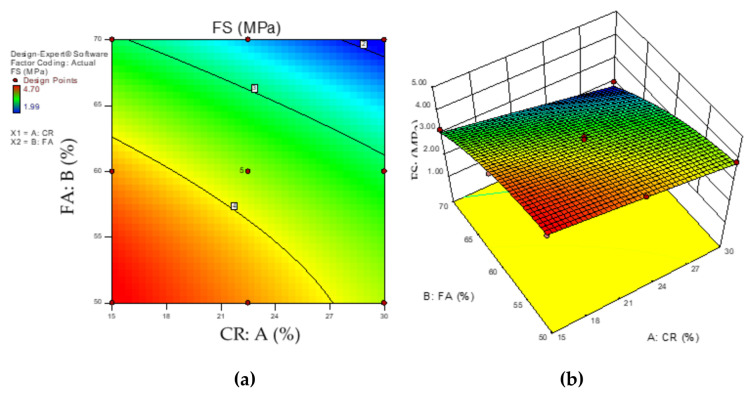
HVFA–CRC 2D and 3D response surface diagrams for FS.

Figure 12a,b show the response surface graphs (2D and 3D) for the influence of the interaction between the CR and HVFA on the flexural strength. As depicted by the red regions of the graphs, the lower values of the input factors yielded higher flexural strengths. As the content of the CR and FA increased, the flexural strength reduced, as shown by the green region (intermediate FS) to the blue region (lowest FS). To produce an HVFA–CRC with a significant FS, the level of replacement for CR and FA should not go beyond the regions bounded by 60–65% and 15–27%, respectively.

### 3.4. HVFA–CRC Splitting Tensile Strength

**Figure 13:**
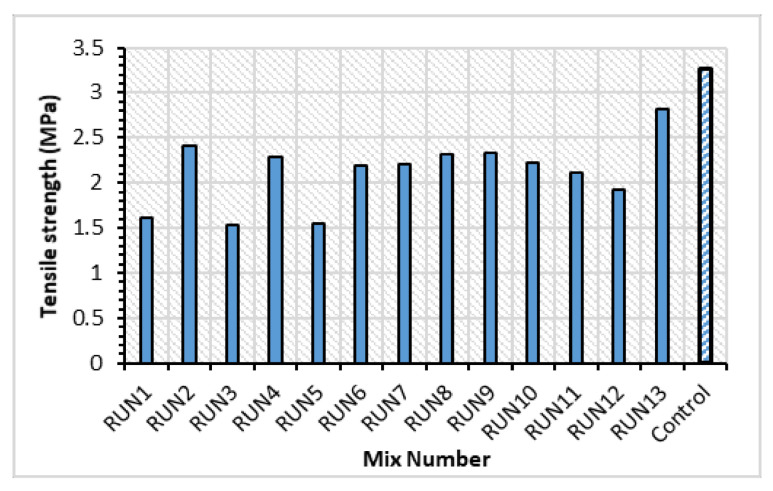
Tensile strength of the HVFA–CRC at 28 days.

**Figure 14:**
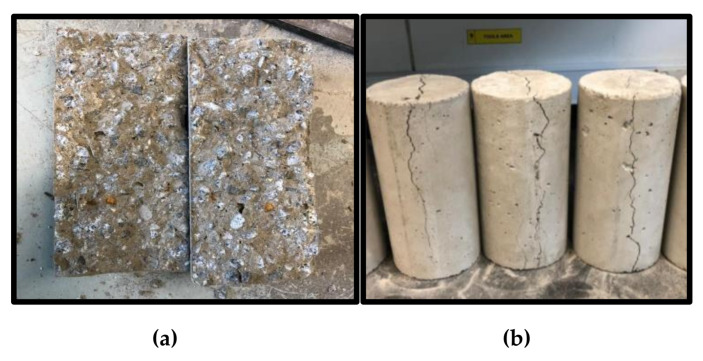
Splitting tensile test samples at failure: (**a**) control and (**b**) HVFA–CRC samples.

The HVFA–CRC splitting tensile strength test result is shown in Figure 13. The splitting tensile strength was negatively affected by both the CR and HVFA incorporation. The tensile strength was inversely proportional to the replacement levels of the input factors (CR and FA). Compared with the control mix, the strength of the concrete with the lowest CR and HVFA replacement (RUN15) has reduced by 13.5%. Mix RUN3 had the lowest tensile strength (1.53 MPa) by virtue of having the highest CR and FA replacement. This is in line with the work of Fauzan et al. [19]. The decreasing strength of the HVFA–CRC was attributed to the lack of proper bonding between the CR and the hardened matrix and to the reduced cement content due to the HVFA replacement. It was, however, noticed that mixes having a lower CR and FA content experienced more brittle failure. Hence, the higher content of CR and FA led to more energy absorption and ductile failure. As shown in Figure 14a, the control sample split into two parts at failure (typical brittle failure), while the other samples with CR and HVFA remained intact with only longitudinal cracks at failure, as depicted in Figure 14b.

**Figure 15:**
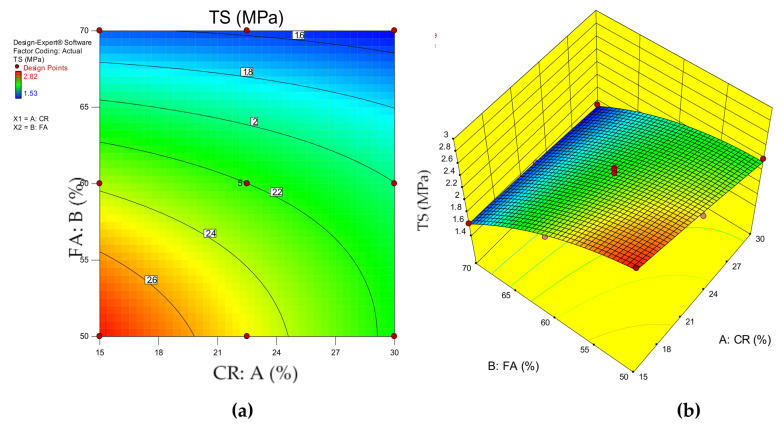
HVFA–CRC 2D and 3D response surface diagrams for the STS.

The behavior of the HVFA–CRC due to the effect of the CR and FA replacement values is shown visually using the 2D contour and 3D response surface plots in Figure 15a,b, respectively. The graphs depict how the interaction of the independent variables affected the response (TS).

## 4. Response Surface Models and ANOVA Validation

The response prediction models were developed using the RSM and their adequacy was verified using analysis of variance (ANOVA). A response model can take the form of a linear or quadratic polynomial, as shown in Equations (1) and (2), respectively [34,35]:

(1) $$y=\beta_{0}+\beta_{i}x_{i}+\beta_{2}x_{2}+\beta_{n}x_{n}+\epsilon$$

(2) $$y=\beta_{0}+\overset{k}{\underset{i=1}{\sum }}\beta_{i}x_{i}+\overset{k}{\underset{i=1}{\sum }}\beta_{ii}x_{i}^{2}+\overset{k}{\underset{j=2}{\sum }}\overset{j=1}{\underset{i=1}{\sum }}\beta_{ij}x_{i}x_{j}+\epsilon \;$$

where *y* signifies the desired response, $\beta_{0}$ is the regression coefficient for the constant term, while the other regression coefficients are $\beta_{i}$ for the linear term, $\beta_{ii}$ for the quadratic term, and $\beta_{ij}$ for the interaction of the $x_{i}$ and $x_{j}$ factors. The number of factors is represented with *k* and ϵ is the random error.

The model equations (in coded terms) developed for the three responses (compressive, flexural, and tensile strengths of HVFA–CRC) are presented in Equations (3)–(5). All the responses were fitted with quadratic models, as shown:

(3) $$CS=+23.87-2.32\;*\;A-6.44*\;B+0.14*\;AB-0.79*\;A^{2}-3.08*\;B^{2}$$

(4) $$FS=+3.70-0.55\;*\;A-0.93*\;B-0.087*\;AB-0.016*\;A^{2}-0.33*\;B^{2}$$

(5) $$TS=+2.20-0.19\;*\;A-0.46*\;B+0.13*\;AB-0.014*\;A^{2}-0.17*\;B^{2}$$

where CS is the compressive strength (MPa), the flexural strength is FS (MPa), TS is the tensile strength (MPa), A is the CR replacement (%), and B is the HVFA replacement (%).

The adequacy of the developed response models was checked using ANOVA, the summary of which is presented in Table 4. The analysis was performed with a 95% confidence interval (5% level of significance). Hence, all models and model terms with a probability below 0.05 were considered statistically significant [36]. Therefore for CS, FS, and TS, the significant model terms were A, B, and B^2^. Furthermore, AB was a significant model term for TS. To ensure the fitness of the models to the data, the lack of fit values must be insignificant [37]. The lack of fit F-values for the models were 2.87, 2.54, and 1.04 for the CS, FS, and TS, respectively. These values signified that the lack of fit values for the models were insignificant compared to the pure error.

Another measure for the strength of a model is the coefficient of determination (R^2^). The R^2^ is a measure of how close the data is to the fitted model. Generally speaking, the higher the R^2^ value (on a scale of 0 to 100%), the better the model fits the data. In this case, the R^2^ values were 97.7%, 95.3%, and 97.3% for CS, FS, and STS, respectively, as presented in Table 5 for the model validation parameters. These high values of the coefficient of determination indicated how well the models fit the data.

In the same vein, the difference between the Adj. R^2^ and Pred. R^2^ should be less than 0.2 for the models to fit [38]. In this case, the difference between these parameters for all the models was less than 0.2, as can be seen from Table 5. Similarly, the signal-to-noise ratio is measured by the adequate precision value (Adeq. Precision), and a value of more than 4 is required [30]. In this case, the Adeq. Precision values were 25.116, 19.018, and 23.841 for the CS, FS, and STS, respectively. These values show that there was a good signal and the models were strong and can be used to navigate the design space.

**Figure 16:**
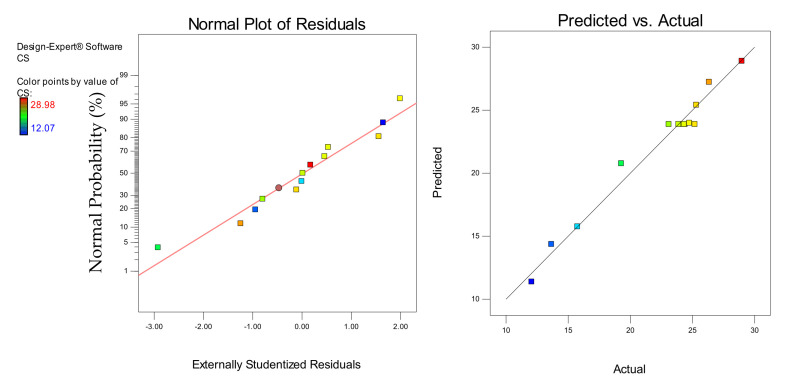
Normal plot of residuals and predicted vs. actual plots for the CS

**Figure 17:**
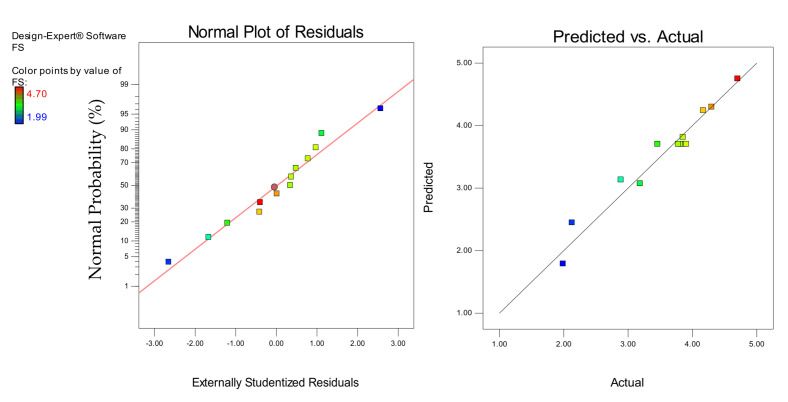
Normal plot of residuals and predicted vs. actual plots for the FS.

**Figure 18:**
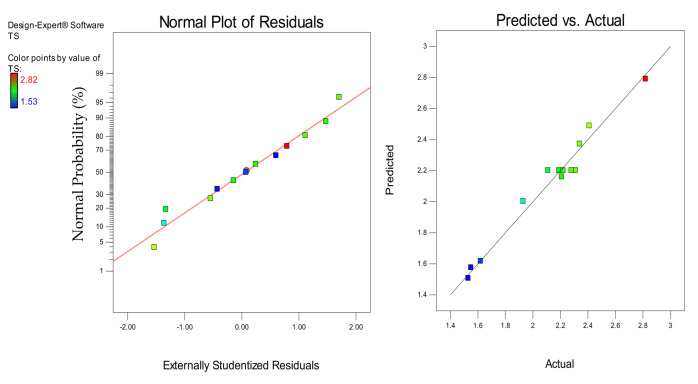
Normal plot of residuals and predicted vs. actual plots for the TS.

Models diagnostics was performed using the normal plots of residuals and the actual versus predicted graphs shown in Figure 16, Figure 17 and Figure 18 for the CS, FS, and TS, respectively. In all cases, the linearity of the data points around the line of fit gave a good sign for the models’ accuracy in predicting the responses. For the actual versus predicted graphs, the alignment of the points around the fitted line for all the responses shows how close the predicted responses were to the actual responses. Similarly, in the normal plots of residuals for all the responses, the linear distribution of the data points around the line of fit shows that the models were strong and the error terms were normally distributed [27].

## 5. Multi-Objective Optimization (MO)

MO (or multi-response optimization) is a method that is used to determine the optimal amount (level) of the input variables to concurrently improve two or more responses. Most real-life optimization situations involve the need to strike a balance between more than one (often conflicting) objectives [39]. The optimization is done by assigning targets for the input factors and the responses and level of significance to attain balanced objective functions. The optimization is measured using the “desirability value,” which is measured on a scale of 0 to 1 (0 ≤ d ≤ 1) [24].

**Figure 19:**
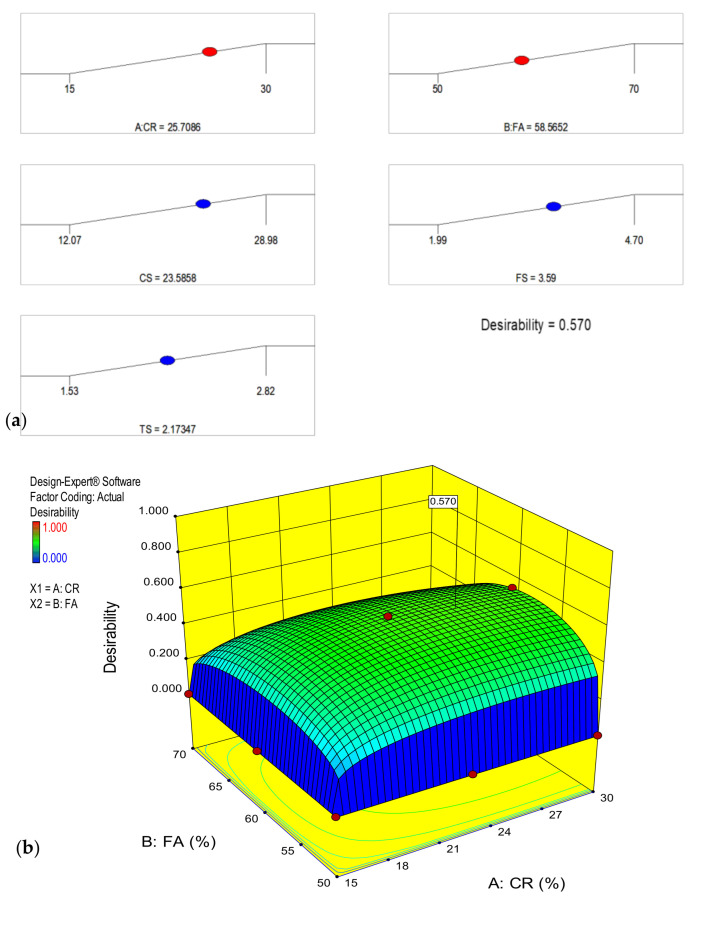
(**a**) Optimization ramp (**b**) 3D response surface plot for the optimization.

In this research, the optimization criteria and the result are shown in Table 6. The target for the input factors was set to “maximize” such that the highest possible amount of the CR and HVFA could be utilized to attain a structural concrete. The objective of the optimization was to maximize all three responses and the goal was set accordingly. When the optimization was performed, the RSM generated 25.7% and 58.6% as the optimal levels of the CR and FA to achieve a result of 23.58 MPa, 3.59 MPa, and 2.2.17 MPa for the CS, FS, and STS, respectively, at a desirability value of 57%. The optimization result is shown in Figure 19a,b as optimization ramp and 3D response diagrams, respectively.

The optimization result was experimentally validated by producing HVFA–CRC samples using the RSM-generated CR and HVFA replacements. Samples that were used to determine the CS, FS, and STS were cast, cured for 28 days, and tested. The averages of the results are shown in Table 7. The percentage error between the predicted and the experimental responses was calculated using Equation (6) and the results are presented in Table 7. For all the responses, the values of the error were found to be less than 5%, which shows the accuracy of the developed response predictive models.

(6) $$\delta =\left|\frac{\vartheta_{E}-\vartheta_{P}}{\vartheta_{P}}\right|\times 100\%\;$$

where δ is the percentage error, $\vartheta_{E}$ is the experimental value, $\vartheta_{P}$ predicted value.

## 6. Conclusions

The following conclusions were drawn at the end of the research:

1. An increase in the slump by 80–200% was observed by mixes having 50–70% HVFA content at the same CR content.
2. CR affected the workability of the concrete via a 16.6–50% reduction in the slump between mixes having 15–30% CR at a 70% HVFA replacement level.
3. The mechanical strengths of the concrete were negatively affected by the increase in CR and HVFA replacements. Nevertheless, a 28-day strength of more than 20 MPa was attained by many of the mixes with CR and HVFA replacements of less than 22.25 and 60%, respectively. Conversely, with higher CR and HVFA replacements, the ductility was enhanced, leading to better deflection capacity, energy absorption, and change in the failure mode from brittle to ductile.
4. Response predictive models were developed and validated with a high R^2^ of 97.74%, 95.26%, and 97.36 for the CS, FS, and STS, respectively. Multi-objective optimization performed yielded optimal values of 15% and 50% for CR and HVFA, respectively, to achieve 28.89 MPa, 4.75 MPa, and 2.79 MPa for the CS, FS, and STS, respectively, at a desirability value of 99%. Experimental validation showed a high level of agreement between the predicted and the experimental values with a percentage error of less than 5%.
5. The optimization results showed that 25.7% CR replacement of fine aggregate and 58.6% HVFA replacement of cement were the optimal values of the input factors that will produce HVFA–CRC that is suitable for structural applications.

The utilization of high amounts of CR and HVFA together to make the structural concrete produced in this work is a positive result that can help in tackling the waste disposal and environmental degradation problems, which will lead to achieving environmental sustainability. However, the models were developed for a low calcium class F FA and CR that had the properties specified in this research work. Any deviation from the materials’ properties may lead to an inaccurate response prediction.

## Figures and Tables

**Table 1:** Chemical composition of the OPC and FA used.

Oxide	CaO	SiO_2_	Fe_2_O_3_	Al_2_O_3_	K_2_O	MgO	SO_3_	P_2_O_5_	TiO_2_	MnO	ZnO	SrO	CuO	As_2_O_3_
OPC	82.10	8.59	3.18	2.00	0.72	0.62	2.78	0.56	0.17	0.15	0.30	0.30	0.30	0.20
FA	6.57	62.40	9.17	15.30	1.49	0.77	0.65	1.23	1.32	0.77	0.03	0.19	0.02	0.01

**Table 2:** RSM generated mixes and quantities of materials used.

Mix/Experimental Runs	Input Factors			Materials (kg)					
	CR (%)		FA (%)	CR	FA	Cement	Fine Aggregate	Coarse Aggregate	Water
RUN1	15		70	0.81	7.88	3.38	17.03	39.28	4.72
RUN2	22.5		50	1.22	5.63	5.63	15.53	39.28	4.72
RUN3	30		70	1.63	7.88	3.38	14.03	39.28	4.72
RUN4	22.5		60	1.22	6.75	4.5	15.53	39.28	4.72
RUN5	22.5		70	1.22	7.88	3.38	15.53	39.28	4.72
RUN6	22.5		60	1.22	6.75	4.5	15.53	39.28	4.72
RUN7	30		50	1.63	5.63	5.63	14.03	39.28	4.72
RUN8	22.5		60	1.22	6.75	4.5	15.53	39.28	4.72
RUN9	15		60	0.81	6.75	4.5	17.03	39.28	4.72
RUN10	22.5		60	1.22	6.75	4.5	15.53	39.28	4.72
RUN11	22.5		60	1.22	6.75	4.5	15.53	39.28	4.72
RUN12	30		60	1.63	6.75	4.5	14.03	39.28	4.72
RUN13	15		50	0.81	5.63	5.63	17.03	39.28	4.72
Control	-		-	0	0	11.26	20.03	39.28	4.72

**Table 3:** Results of fresh and hardened properties of the HVFA–CRC.

Run	A: CR (%)	B: FA (%)	Slump (mm)	CS (MPa)	FS (MPa)	Deflection (mm)	STS (MPa)
RUN1	15	70	45	15.75	3.2	3.30	1.62
RUN2	22.5	50	10	26.35	4.3	2.60	2.41
RUN3	30	70	30	12.07	2.0	3.60	1.53
RUN4	22.5	60	20	24.40	3.9	2.80	2.28
RUN5	22.5	70	35	13.66	2.1	3.51	1.55
RUN6	22.5	60	20	23.11	3.9	2.78	2.19
RUN7	30	50	10	24.76	3.9	2.82	2.21
RUN8	22.5	60	22	23.89	3.5	3.00	2.31
RUN9	15	60	25	25.32	4.2	2.67	2.34
RUN10	22.5	60	19	24.33	3.8	2.78	2.22
RUN11	22.5	60	21	25.20	3.8	2.80	2.11
RUN12	30	60	15	19.28	2.9	3.45	1.93
RUN13	15	50	15	28.98	4.7	2.56	2.82

**Table 4:** Result of the ANOVA.

Response	Source	Sum of Squares	df	Mean Square	F-Value	*p*-Value > F	Significance
Compressive Strength (MPa)	Model	319.67	5	63.93	60.66	<0.0001	Yes
	A–CR	32.39	1	32.39	30.73	0.0009	Yes
	B–FA	248.46	1	248.46	235.75	<0.0001	Yes
	AB	0.073	1	0.073	0.069	0.8001	No
	A^2^	1.72	1	1.72	1.63	0.2419	No
	B^2^	26.28	1	26.28	24.94	0.0016	Yes
	Residual	7.38	7	1.05			
	Lack of Fit	5.04	3	1.68	2.87	0.1673	No
	Pure error	2.34	4	0.59			
Flexural Strength (MPa)	Model	7.38	5	1.48	28.13	0.0002	Yes
	A–CR	1.84	1	1.84	35.15	0.0006	Yes
	B–FA	5.14	1	5.14	97.92	< 0.0001	Yes
	AB	0.030	1	0.030	0.58	0.4726	No
	A^2^	6.959 × 10^−4^	1	6.959 × 10^−4^	0.013	0.9116	No
	B^2^	0.30	1	0.30	5.76	0.0474	Yes
	Residual	0.37	7	0.052			
	Lack of Fit	0.24	3	0.080	2.54	0.1947	No
	Pure error	0.13	4	0.032			
Splitting Tensile Strength (MPa)	Model	1.62	5	0.32	51.68	<0.0001	Yes
	A–CR	0.21	1	0.21	32.71	0.0007	Yes
	B–FA	1.25	1	1.25	199.30	<0.0001	Yes
	AB	0.068	1	0.068	10.77	0.0135	Yes
	A^2^	5.124 × 10^−4^	1	5.124 × 10^−4^	0.082	0.7834	No
	B^2^	0.079	1	0.079	12.51	0.0095	Yes
	Residual	0.044	7	6.278 × 10^−3^			
	Lack of Fit	0.019	3	6.422 × 10^−3^	1.04	0.4652	No
	Pure error	0.025	4	6.170 × 10^−3^			

**Table 5:** Model validation parameters.

Model Validation Parameters	Responses		
	Compressive Strength (MPa)	Flexural Strength (MPa)	Split Tensile Strength (MPa)
Std. Dev.	1.03	0.23	0.079
Mean	22.08	3.54	2.12
C.V.%	4.65	6.47	3.74
PRESS	42.73	2.16	0.18
-2Log Likelihood	29.53	−9.47	−37.07
R^2^	0.9774	0.9526	0.9736
Adj. R^2^	0.9613	0.9187	0.9548
Pred. R^2^	0.8693	0.7215	0.8928
Adeq. Precision	25.116	19.018	23.841
BIC	44.92	5.92	−21.68
AIC	55.53	16.53	−11.07

**Table 6:** Optimization criteria and result.

Factors		Variable (Input Factors)		Response (Output Factors)		
		CR *(%)*	FA *(%)*	CS (MPa)	FS (MPa)	STS (MPa)
Value	Minimum	15	50	12.07	1.99	1.53
	Maximum	30	70	28.98	4.70	2.82
Goal		Maximize	Maximize	Maximize	Maximize	Maximize
Optimization result		25.7	58.6	23.58	3.59	2.17
Desirability		0.57 (57%)				

**Table 7:** Experimental validation.

Response	$\mathrm{Predicted}\;(\vartheta_{P})$	$\mathrm{Experimental}\;(\vartheta_{E})$	Error, δ (%)
Compressive strength (MPa)	23.58	22.80	3.3
Flexural strength (MPa)	3.59	3.71	3.3
Splitting tensile strength (MPa)	2.17	2.26	4.1

## Data Availability

The data presented in this study are available in [Utilization of Crumb Rubber and High-Volume Fly Ash in Concrete for Environmental Sustainability: RSM-Based Modeling and Optimization].
